# A Study on Job Satisfaction and Burnout Levels of Veterinarians in Türkiye

**DOI:** 10.1002/vms3.70500

**Published:** 2025-07-18

**Authors:** Seda Çavuş Alan, Rahşan Özen, Abdullah Özen, Abdullah Eryol

**Affiliations:** ^1^ Department of History of Veterinary Medicine and Deontology, Faculty of Veterinary Medicine Kafkas University Kars Türkiye; ^2^ Department of History of Veterinary Medicine and Deontology Faculty of Veterinary Medicine, Firat University Elazig Türkiye; ^3^ Department of History of Veterinary Medicine and Deontology, Faculty of Veterinary Medicine Atatürk University Erzurum Türkiye

**Keywords:** burnout | job satisfaction | veterinary | work‐associated

## Abstract

**Background:**

Burnout is among the most common problems faced in the veterinary profession, which is in constant contact with animals and people. It is already known that job satisfaction is the leading factor that affects professional burnout.

**Objectives:**

The present study aimed to determine the job satisfaction and burnout levels of veterinarians working in Türkiye, to uncover the factors that might affect job satisfaction and burnout levels and to understand whether there is a relationship between job satisfaction and burnout levels.

**Methods:**

This cross‐sectional study was conducted with 2276 veterinarians using the Maslach Burnout Inventory and the Minnesota Satisfaction Questionnaire.

**Results:**

It was found that participants who were older, who had more years of experience in the profession, who were parents and who had higher incomes had lower burnout levels. Job satisfaction was mainly influenced by income level. Working in academia was found to be associated with lower levels of burnout, whereas working in a municipality was associated with higher levels of burnout. It was also found that having chosen the profession willingly and thinking of wanting to be a veterinarian again if given the chance was associated with lower levels of burnout and higher job satisfaction.

**Conclusion:**

It has been concluded that job satisfaction and burnout levels in veterinarians affect each other.

## Graphical abstract

Being older, having more years of professional experience, being a parent and having a higher income are associated with lower levels of burnout among veterinarians. Income level emerged as the most influential factor in increasing job satisfaction. Additionally, the field of study significantly affects burnout levels.

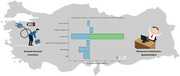

## 1 Introduction

The concepts of professional burnout and job satisfaction are intertwined and complementary for individuals who spend a significant portion of their lives at work. The concept of burnout was introduced in 1970 and is generally used to describe the state of exhaustion that occurs as a result of failure to achieve success against stress factors, wear and tear, low resistance and internal demands that cannot be covered. Burnout, which occurs more frequently in individuals with professions that require face‐to‐face communication, is a syndrome with many different dimensions such as personal burnout, work‐associated burnout, client‐related burnout, withdrawal, physical exhaustion, cognitive weariness, lack of enthusiasm towards the job, indolence and guilt (Gil‐Monte 2012; Demerouti and Bakker 2008; Kristensen et al. 2005). This concept was addressed from different aspects in different studies and still continues to be dealt with. Three dimensions came to the fore in the study conducted by Maslach and Jackson to develop a burnout scale (Maslach and Jackson 1981) as ‘Emotional Exhaustion’, ‘Depersonalization’ and ‘Lack of Personal Accomplishment’.

The main factors that affect burnout are work life and, therefore, job satisfaction (McNeese‐Smith and Crook 2003). Job satisfaction, which is expressed as the general attitude towards work, is defined as the balance of what is expected from the work done and what the job offers. Employees enjoy their work to the extent that their needs are covered, feel happy and peaceful, do their job with pleasure and, therefore, work more effectively and efficiently (Hackman and Oldham 1976; Gazi et al. 2024). Dissatisfaction occurs when the employee's desires and the characteristics of the job do not match. This might cause a negative attitude towards work. This, defined as job dissatisfaction, results in disinterest in work, low performance, indiscipline and constant job changes (Locke 1969; Eğinli 2009). Factors such as working conditions, workload, lack of appreciation and inadequate wages might cause dissatisfaction in the person towards the job, and this might affect burnout (McNeese‐Smith and Crook 2003; Andela 2020). Veterinarians are generally hard‐working, dedicated healthcare professionals who are passionate about their work. However, when faced with high levels of workplace stress and overwhelming demands, these veterinarians may feel like they are about to collapse 1 day (Steffey et al. 2023).

Previous studies that were conducted on different healthcare staff have examined job satisfaction and burnout levels. Studies conducted during the COVID‐19 pandemic reported that doctors (Alrawashdeh et al. 2021) and nurses (Heidari et al. 2022) experienced high levels of burnout and low job satisfaction due to the high demands of the pandemic. In a study conducted in China, it was reported that psychiatrists experienced high rates of burnout and job dissatisfaction (Yao et al. 2021). Sarabi et al. (2020) reported that emotional exhaustion in healthcare staff working in rural areas had a significant effect on overall job satisfaction levels and that an increase in emotional exhaustion levels caused a decrease in job satisfaction. It was argued in recent years that burnout is also common among veterinary professionals who are in constant contact with animals and people (Andela 2020; Hatch et al. 2011; Hanrahan et al. 2018; Kogan et al. 2020; Brscic et al. 2021) and that low job satisfaction is among the main reasons for leaving work in the veterinary profession (Kersebohm et al. 2017; Limb 2018). It was also reported that job satisfaction also affects stress, burnout and suicide cases (Kersebohm et al. 2017; Limb 2018; Clise et al. 2021; Dalum et al. 2022). It is considered that the increasing number of suicide cases among veterinarians, especially in recent years, not only causes personal harm but also damages the image of the profession, thus ‘demonizing’ the profession, and this causes newly graduated veterinarians to have a prejudiced approach towards business life (Clise et al. 2021).

Factors that are already known to affect burnout in veterinary medicine include increased clinical workload, extended working hours, expectations of animal owners, workspace, career concerns, the addition of administrative duties and worsening financial situation (Hatch et al. 2011; Volk et al. 2018; Dawson and Thompson 2017; Gardner and Hini 2006; Adin et al. 2021; Ouedraogo et al. 2021; Hayes et al. 2020). Burnout, which has a gradual progression, deepens if symptoms are not recognized, and unexpected sudden reactions might develop. A veterinarian experiencing burnout might experience disruptions in fulfilling related obligations to their patients, patient owners, other service providers they work with and society. It is argued that this both reduces personal earning potential and causes socioeconomic costs (Hayes et al. 2020; Neill et al. 2022; Foote 2020).

There are studies in the literature examining the job satisfaction (Kersebohm et al. 2017; Limb 2018; Agrawal and Agrawal 2014) and burnout levels (Hatch et al. 2011; Gardner and Hini 2006; Hansez et al. 2008) of veterinarians in various countries. A study conducted in the United States reported that veterinarians had low job satisfaction levels, and only 41% said that they would recommend their profession to a friend or family member (Volk et al. 2018). Another study conducted in the United Kingdom reported that 37% of veterinarians were considering leaving their jobs, and one‐quarter were not sufficiently committed to their careers (Limb 2018). Another study that was conducted with veterinarians on duty showed that long shift hours had a negative impact on job satisfaction and personal relationships (Kogan et al. 2020). This study aimed to determine the job satisfaction and burnout levels of veterinarians working in Türkiye, uncover the factors that might have effects on job satisfaction and burnout levels and understand whether there is a relationship between job satisfaction and burnout levels.

## 2 Materials and Methods

### 2.1 Participants

The population of the study consisted of veterinarians working in Türkiye. The total number of veterinarians in Türkiye was reported as approximately 40,000 by the Turkish Veterinary Association (Türk Veteriner Hekimleri Birliği 2022). Although it is recommended that the sample should be between 378 and 381 (Krejcie and Morgan 1970) when this number is taken into account, the study aimed to reach the largest possible number of participants to increase the representativeness of the sample, and a questionnaire was administered to 2276 people. The Relational Screening Model, which is one of the quantitative research methods and aims to reach a general judgment about the universe, was employed in this cross‐sectional study.

### 2.2 Data Collection

The data were collected with an online questionnaire (Google Form). Participants joined the study via a link, which was shared in WhatsApp groups that only included veterinarians. When they clicked on the link, they reached the study. An informative text explaining the study and the right to withdraw was added to the first page of the questionnaire. An option was added below this text stating that the participants approved participating in the study, and only those who gave this approval could see the questionnaire questions. In this way, the study was ensured to be based on volunteering. Additionally, when preparing a survey in Google Forms, the ‘necessary’ button in the bottom right corner for each question was activated. The system did not allow the survey to be finished until all questions were answered, thus preventing missing data. As the inclusion criterion for the study was ‘being a veterinarian’, those who were not veterinarians were excluded from the study. The data were obtained between 21.06.2023 and 21.09.2023. The questionnaire form used as a data collection tool consisted of three sections: Section 2.2.1, Section 2.2.2 and Section 2.2.3. The questionnaire form can be accessed in the ‘Supporting Information’ section at the end of the article.

#### 2.2.1 Personal Data

The purpose of this section, prepared by the researchers, was to obtain data on the sociodemographic data of the participants and some other independent variables. When classifying the ‘field of study’ heading, groups with 10 or fewer participants were evaluated under the category of ‘Other’. The category of ‘Other’ consisted of the following fields of study: experimental animals, feed industry, equine medicine, pet training, pet hotel management, non‐governmental organizations, military, wildlife, nature conservation and national parks, retirees and those who were not working.

#### 2.2.2 Minnesota Satisfaction Questionnaire

It was developed by Weiss et al. (1967) and was translated into Turkish by Baycan (1985). Its validity and reliability studies were conducted. The Cronbach alpha reliability coefficient of the scale was calculated as 0.77. The scale consists of 20 items presented with a 5‐point Likert type. The highest score that might be obtained from the scale is 100, and the lowest score is 20. Scores of 25 and below indicate low job satisfaction, scores between 26 and 74 indicate normal job satisfaction, and scores above 75 indicate high job satisfaction (Weiss et al. 1967; Atabey 2012).

#### 2.2.3 Maslach Burnout Inventory

The ‘Maslach Burnout Inventory’ was developed by Maslach and Jackson (1981) consisting of a total of 22 items, and its Turkish reliability and validity studies were conducted in 1992 (Ergin 1992). Although the items were presented with a 7‐point Likert‐type scale in the original scale, they were presented with a 5‐point Likert‐type scale in the Turkish validity and reliability study (Ergin 1992). Although the participants’ burnout levels were tried to be measured with a set of 22 items in both the original scale and the Turkish version, these studies argued that burnout is a syndrome with many components and there is no claim that the total scores obtained from this scale (0–88 scores) alone indicate the level of burnout. No cut‐off value was employed to convert these scores into a judgment defining the level of burnout.

### 2.3 Data Analysis

Before using the principal component analysis (PCA), Bartlett's test of sphericity was used to determine the adequacy of the data for analysis because the scales used in the study were applied to a new sample, and the Kaiser–Meyer–Olkin (KMO) test was used to measure the sample adequacy (*p* < 0.001 and KMO ≥ 0.5 and show the adequacy of the data for use in the PCA). PCA with varimax rotation was used to extract key factors. Cronbach's alpha coefficient was used for reliability, and the JAMOVI 2.2.5 (The Jamovi Project, Sydney, Australia) package program was used to analyse the data. Normal distribution was examined using descriptive statistics (standard deviation, mean, median, skewness, kurtosis), graphical methods (histogram, boxplot and QQ plots) and tests for normality (Kolmogorov–Smirnov), and it was found that the data were normally distributed for both scales. For pairwise group comparisons for variables, the independent *t*‐test was used, and for comparisons of three or more groups, the ANOVA test was used. In the ANOVA test, the homogeneity of variances was examined with the Homogeneity of Variance Test to identify different groups. Duncan and Bonferroni's T2 tests were performed in groups with homogeneous variances, and Games‐Howell and Tamhane's T2 tests were performed on the basis of the sample numbers in the subgroups in groups with inhomogeneous variances. Multiple linear regression was performed to examine the effect of demographic variables simultaneously. In this analysis, quantitative data were analysed by using the SPSS version 22.0 software. The results were evaluated at a 95% confidence interval.

## 3 Results

**FIGURE 1:**
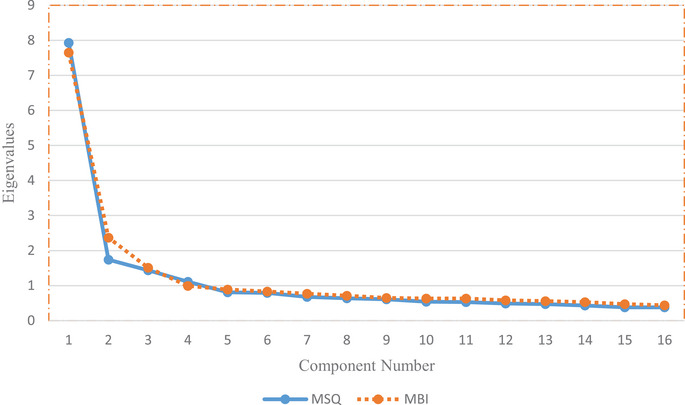
Scree plot chart of components in Minnesota Satisfaction Questionnaire (MSQ) and Maslach Burnout Inventory (MBI).

The responses of 2276 participants who participated in the study were analysed. PCA test, Bartlett's Test of Sphericity and KMO Test were used to determine the factor structure of MBI and MSQ scales on veterinarians, and data on the suitability of the sample for factor analysis are given in Table 1. As a result of PCA, a scree plot chart was examined, and four factors with an eigenvalue above 1, explaining 61.2% of the variance for MSQ, and three factors explaining 52.5% of the variance for MBI were formed (Figure 1). Cronbach's alpha values for MSQ and MBI were 0.91 and 0.90, respectively (Table 1).

**TABLE 1:** Principal component analysis results of the scales.

Variables	Cronbach's alpha	Bartlett's test of sphericity	Kaiser–Meyer–Olkin	Percentage of total variance
**MSQ**	0.91	*χ* ^2^: 23,000	0.92	61.2
		Df: 190		
		*p* < 0.001		
**MBI**	0.90	*χ* ^2^: 22,415		
		Df: 231	0.93	52.5
		*p* < 0.001		

Abbreviations: MBI, Maslach Burnout Inventory; MSQ, Minnesota Satisfaction Questionnaire.

When sociodemographic data were evaluated, it was found that the majority of the participants were male (75%), married (77.8%) and had children (69%). More than half (57.5%) worked in the Ministry of Agriculture and Forestry. The vast majority (82.8%) said that they chose the veterinary profession willingly, while the rate of those who said they would choose to be a veterinarian again if given the chance was determined as 52.2%. Moreover, more than a third of the participants defined their monthly income as low (37.4%), whereas 57.5% defined it as medium level (Tables 2 and 3). The total scores calculated based on the veterinarians’ responses were 62.45 for the MSQ and 35.79 for the MBI.

**TABLE 2:** Changes in the mean scores of the scales as a result of ANOVA according to sociodemographic characteristics.

Variables	Groups	*N* (%)	MSQ		*p* and *F* value	MBI		*p* and *F* value
			Mean	SD		Mean	SD	
**Age**	22–30 age	397 (17.5)	62.62^b^ ^c^	14.58	*p* < 0.0001 *F* = 9.61	40.65^a^	13.25	*p* < 0.0001 *F* = 56.34
	31–39 age	902 (39.6)	60.91^c^	13.82		37.78^b^	12.84	
	40–48 ages	559 (24.6)	62.42^bc^	13.95		34.24^c^	12.15	
	49–57 age	319 (14)	64.73^b^	14.35		30.30^d^	12.48	
	58 and over age	99 (4.3)	68.63^a^	13.41		24.62^e^	12.47	
**Daily working hours**	Less than 8 h	232 (10.2)	63.55	14.22	*p* = 0.144 *F* = 1.93	33.02^c^	12.75	*p* < 0.0001 *F* = 14.51
	8–10 h	1400 (61.5)	61.56	14.11		35.50^b^	12.96	
	11–12 h	229 (10.1)	61.64	15.33		40.75^a^	14.16	
	Flexible	415 (18.2)	65.28	13.19		35.60^b^	13.53	
**Years of professional experience**	0–5 years	417 (18.3)	62.45^b^	14.61	*p* < 0.0001 *F* = 7.18	40.09^a^	13.51	*p* < 0.0001 *F* = 45.87
	6–10 years	455 (20.0)	60.05^c^	13.70		40.27^a^	12.73	
	11–15 years	519 (22.8)	61.91^bc^	13.90		35.54^b^	12.42	
	16–20 years	298 (13.1)	62.02^bc^	14.37		33.84^bc^	11.98	
	21–25 years	237 (10.4)	64.00^ab^	13.77		32.87^c^	11.93	
	26 years and over	350 (15.4)	65.70^a^	13.98		28.85^d^	12.99	
**Field of study**	Pet clinic	255 (11.2)	66.29^ab^	14.14	*p* < 0.0001 *F* = 23.56	40.53^ab^	13.41	*p* < 0.0001 *F* = 16.50
	Farm animal medicine	284 (12.5)	65.13^b^	12.76		38.54^abc^	12.72	
	Ministry of Agriculture and Forestry	1311 (57.5)	59.75^c^	13.87		35.19^bcd^	12.81	
	Academic veterinarian	188 (8.3)	71.11^a^	12.24		28.29^e^	12.26	
	Municipality	56 (2.5)	57.28^c^	11.58		42.03^a^	11.84	
	Occupational veterinarians	56 (2.5)	59.42^c^	16.32		37.58^abc^	16.14	
	Poultry	14 (0.6)	66.35^ab^	14.06		33.85^cd^	12.07	
	Pharmaceutical industry	20 (0.9)	71.70^a^	16.83		29.90^de^	14.27	
	Other	92 (4.0)	66.69^ab^	12.28		34.82^cd^	13.59	
**Income level**	Low	852 (37.4)	56.46^c^	13.86	*p* < 0.0001 *F* = 186.44	39.13^a^	12.85	*p* < 0.0001 *F* = 64.12
	Middle	1308 (57.5)	65.06^b^	12.69		34.42^b^	12.85	
	High	116 (5.1)	77.06^a^	12.30		26.75^c^	14.31	

Note: Values sharing the same superscript letter are not significantly different (*p* > 0.05); different letters indicate significant differences (*p* < 0.05) based on post‐hoc analysis.

Abbreviations: df, degree of freedom; MBI, Maslach Burnout Inventory; MSQ, Minnesota Satisfaction Questionnaire; N, number; P, probability; SD, standard deviation.

**TABLE 3:** Changes in the mean scores of the scales as a result of the independent *t*‐test according to sociodemographic characteristics.

			MSQ				MBI			
Variables	Groups	*N* (%)	Mean ± standard deviation	*T*	df	*p*	Mean ± standard deviation	*T*	df	*p*
**Gender**	Female	569 (25.0)	63.00 ± 14.71	1.04	931.82	0.299	36.96 ± 13.90	2.42	2274	0.015
	Male	1707 (75.0)	62.27 ± 13.96				35.40 ± 13.05			
**Marital status**	Married	1771 (77.8)	62.67 ± 14.01	1.40	2274	0.160	34.80 ± 13.02	−6.71	2274	<0.0001
	Single	505 (22.2)	61.67 ± 14.62				39.26 ± 13.62			
**Parenthood**	Yes	1570 (69.0)	63.06 ± 13.87	3.08	2274	0.002	33.81 ± 12.68	−10.85	2274	<0.0001
	No	706 (31.0)	61.09 ± 14.67				40.19 ± 13.54			
**Did you choose the veterinary profession willingly?**	Yes	1884 (82.8)	62.92 ± 14.10	3.48	2274	0.001	35.52 ± 13.30	−2.17	2274	0.030
	No	392 (17.2)	60.19 ± 14.17				37.11 ± 13.14			
**If you had a choice, would you still want to be a veterinarian?**	Yes	1187 (52.2)	67.39 ± 13.07	18.65	2274	<0.0001	30.98 ± 12.32	−19.48	2274	<0.0001
	No	1089 (47.8)	57.07 ± 13.30				41.04 ± 12.28			

Abbreviations: df, degree of freedom; MBI, Maslach Burnout Inventory; MSQ, Minnesota Satisfaction Questionnaire; N, number; P, probability.

According to the results of the independent *t*‐test and ANOVA conducted in the study, MSQ scores did not significantly differ across groups defined by gender, marital status and daily working hours. When the scores obtained from the scales were evaluated on the basis of the age variable, the MSQ score of the participants in the 58 and over age group was significantly higher (*p* <0.0001) compared to the participants in other categories, whereas the MBI score was lower (*p* < 0.0001) (Table 2). In terms of gender variables, women scored higher than men on the MBI scale (*p* < 0.05). When marital status and MBI scores were compared, it was observed that the single participants scored higher than the married participants (*p* < 0.0001). MSQ scores of those with children were found to be higher than those without children (*p* < 0.01), whereas MBI scores were lower (*p* < 0.0001) (Table 3). On the basis of the variable of years of work in the profession, participants with 26 years and over scored higher on the MSQ scale (*p* < 0.0001) and lower on the MBI scale (*p* < 0.0001) compared to younger participants (Table 2). It was observed that participants who answered *yes* to the question *Did you choose the veterinary profession willingly?* scored significantly higher on the MSQ (*p* < 0.01) and significantly lower on the MBI (*p* < 0.05) compared to the others. Similarly, participants who answered *yes* to the question *If you had a choice, would you still want to be a veterinarian?* scored higher on the MSQ (*p* < 0.0001) and lower on the MBI (*p* < 0.0001) compared to participants who answered *no* (Table 3). When the scale scores of the participants were compared on the basis of their income status, it was found that participants in the high‐income group scored higher on the MSQ scale (*p* < 0.0001) and lower on the MBI (*p* < 0.0001) than participants in the middle‐ and low‐income groups, and participants in the middle‐income group scored higher on the MSQ scale (*p* < 0.0001) and lower on the MBI (*p* < 0.0001) than participants in the low‐income group. When the scores of the participants in the MBI were compared on the basis of their daily working hours groups, it was seen that the participants in the group working between 11 and 12 h received higher scores than those who worked 8–10 h, whereas those working 8–10 h received higher scores than those who worked less than 8 h (*p* < 0.001). When the participants’ field of study scores were evaluated, the participants with the highest MSQ score (*p* < 0.001) and the lowest MBI score were the participants in academia (*p* < 0.0001). Those working in the Ministry of Agriculture and Forestry, municipalities and occupational veterinarians received the lowest scores in terms of job satisfaction (*p* < 0.0001). The participants who received the highest MBI score were those working in municipalities (*p* < 0.001) (Table 2).

The multiple linear regression analysis was applied after the ANOVA and the independent *t*‐test to make controlled analysis of variables. Variables included in the model were age, gender, marital status, parenthood, years of professional experience, daily working hours, income level and field of study. For MBI, the regression model was statistically significant (*F* = 22.05, Adjusted *R* ^2^ = 0.18, *p* < 0.0001). According to the results, age (*p* = 0.0005), parenthood (*p* < 0.0001), years of professional experience (*p* = 0.016), income level (*p* < 0.0001), daily working hours (*p* = 0.011) and field of study (*p* < 0.0001) were significant predictors of burnout. Specifically, older participants, those with children, more professional experience, higher income and working fewer hours or in academic roles were associated with lower levels of burnout. Gender (*p* = 0.346) and marital status (*p* = 0.462) were not found to be significant predictors. Similarly, the regression model evaluating MSQ was statistically significant (*F* = 21.51, Adjusted *R* ^2^ = 0.18, *p* < 0.0001). Among all variables, only income level was found to be a statistically significant predictor of job satisfaction (*p* < 0.0001). Participants with higher income reported significantly higher job satisfaction scores. Age, gender, marital status, parenthood, years of professional experience, daily working hours and field of study were not significantly associated with MSQ scores (*p* > 0.05) (Table 4).

**TABLE 4:** Regression coefficients of variables.

			Maslach Burnout Inventory				Minnesota Satisfaction Questionnaire			
Variable	Reference category	Category	Coefficient	Std. Error	*p* value (category)	*p* value (overall)	Coefficient	Std. Error	*p* value (category)	*p* value (overall)
**Age**	22–30	31–39	0.05	1.18	0.962	0.0005	−1.88	1.26	0.137	0.135
		40–48	−0.65	1.51	0.668		−1.64	1.62	0.310	
		49–57	−3.77	1.92	0.049		−0.12	2.05	0.903	
		58 and over	−8.68	2.316	*p* < 0.001		2.62	2.47	0.288	
**Gender**	Female	Male	−0.57	0.60	0.346	0.346	−0.04	0.66	0.948	0.948
**Marital status**	Married	Single	−0.59	0.81	0.462	0.462	0.39	0.86	0.646	0.646
**Parenthood**	No	Yes	−3.42	0.81	*p* < 0.0001	*p* < 0.0001	2.44	0.86	0.090	0.090
**Years of professional experience**	0–5 years	6–10 years	2.34	1.16	0.043	0.016	−0.80	1.23	0.517	0.643
		11–15 years	−0.22	1.27	0.862		0.65	1.36	0.629	
		16–20 years	−1.37	1.56	0.377		0.69	1.66	0.676	
		21–25 years	−0.98	1.67	0.554		0.75	1.78	0.672	
		26 and over	−1.20	1.96	0.539		−0.21	2.09	0.917	
**Income**	Low	Middle	−4.54	0.54	*p* < 0.0001	*p* < 0.0001	7.43	0.58	*p* < 0.0001	*p* < 0.0001
		High	−12.37	1.25	*p* < 0.001		16.82	1.33	*p* < 0.0001	
**Daily working hours**	Less than 8 h	8–10 h	1.00	0.85	0.23	*0.011*	−0.51	0.91	0.574	0.052
		11–12 h	3.61	1.19	0.002		−3.90	1.27	0.023	
		Flexible	0.68	1.03	0.510		−0.39	1.10	0.722	
**Field of study**	Municipality	Academic veterinarian	−11.76	1.85	*p* < 0.0001	*p* < 0.0001	2.91	1.31	0.026	0.07
		Ministry of Agriculture and Forestry	−7.47	1.64	*p* < 0.0001		−5.66	1.04	0.062	
		Farm animal medicine	−2.42	1.81	0.182		−2.57	1.19	0.032	
		Pet Clinic	−3.58	1.83	0.051		−8.25	1.95	*p* < 0.0001	
		Occupational veterinarians	−4.99	2.27	0.028		−6.17	1.92	0.001	
		Poultry	−7.55	3.60	0.036		−1.78	3.52	0.613	
		Pharmaceutical industry	−9.28	3.14	0.003		2.22	3.00	0.460	
		Other	−6.04	2.04	0.003		−1.07	1.59	0.501	
		***R* ^2^**	0.19				0.19			
		**Adjusted *R* ^2^**	0.18				0.18			
		***F***	22.05				21.51			

## 4 Discussion

In the present study, the job satisfaction and burnout levels of veterinarians, who are in constant communication with both animals and people (animal owners, colleagues, managers, veterinary technicians), were determined. In addition, the relationship between these two states and the factors affecting these states were uncovered. First of all, as the scales were applied to a different group, factor analysis was performed. As a result of PCA, the formation of three factors for MBI was found to be consistent with Maslach and Jackson (1981). Although the MSQ scale was reported as two factors by Weiss et al. (1967), it was found as four factors as a result of PCA in our study. Factor analysis is a method that is sensitive to sample characteristics. The cultural norms, values and perceptions of the sample may affect their responses to the scale items. Therefore, the scale may reveal different factor structures when used in different cultures and samples (Gaskin et al. 2017). The difference in the study may be due to this situation. Then, the internal consistencies of the scales were evaluated, and Cronbach's alpha values for MSQ and MBI were calculated as 0.91 and 0.90, respectively. As Cronbach's alpha values above 0.70 are considered reliable for the scales (Hussey et al. 2025), it was accepted that these two scales were quite reliable. Studies conducted in many countries such as India (Agrawal and Agrawal 2014), Canada (Moore et al. 2014) and England (Limb 2018) show that the job satisfaction levels of veterinarians are generally at moderate levels. When evaluated based on the reference values reported by Weiss et al. (1967), it might be said that the job satisfaction levels of the participants of this study (62.45 points) are at a moderate level, and therefore, the results of this study are parallel to the results of the studies listed above. In this context, it might be argued that veterinary medicine in Türkiye is a profession that meets the expectations of its members at a moderate level.

According to both the independent *t*‐test and regression analysis results of the study, parenthood was identified as a factor associated with lower levels of burnout. Previous studies also showed that veterinarians with children experience fewer symptoms of depression and burnout compared to those without children (Shirangi et al. 2013; Holowaychuk and Lamb 2023) and that the level of professional burnout decreases as the number of children they have increases (Ouedraogo et al. 2021). As a result of these results, it can be said that having children has a protective effect in reducing burnout levels. It might be considered that having a life outside of work and therefore having the chance to find social support might reduce a person's tension, which in turn has a positive effect on stress, burnout and job satisfaction caused by work life. On the other hand, it is also possible to speculate the opposite. It might be associated with parents whose workload increases because of having children moving to positions that require less responsibility in their work life. This information might also be examined in future studies, and more detailed research might be conducted. Some studies have suggested that individuals who are parents experience higher levels of job satisfaction (Frank et al. 1999). However, in the present study, although parenthood was found to be associated with higher job satisfaction in the independent *t*‐test (single‐factor analysis), this relationship was not statistically significant when controlling for other variables in the multiple regression model. This suggests that the observed association might be influenced by other demographic or occupational factors such as income, age or working hours.

In our study, both the ANOVA test and the regression analysis results showed that younger participants had higher levels of burnout than others. The study findings are parallel with the literature (Volk et al. 2018; Dawson and Thompson 2017; Gardner and Hini 2006; Rejula et al. 2003). Cordes and Dougherty (1993) expressed the opinion that the relationship between age and burnout level is associated with some experiences gained in the profession and life rather than just biological age. When the duration of our participants’ work in the profession was examined, it was seen that the burnout levels of the participants with more years of work were lower according to both the regression analysis and the ANOVA test results. The study findings are consistent with the literature (Hatch et al. 2011; Ouedraogo et al. 2021; Shirangi et al. 2013; Fritschi et al. 2009). When these results are evaluated together, this might be explained by the experience gained and the developed work management skills depending on age and length of service, and the ability to cope with stress or stressful situations over the years. The higher levels of burnout in the early years of work life might be because of the difficulties experienced by this group, who have come out of student life, such as adaptation to a new environment (work life), the obligation to repay scholarships and establishing a balance between work and family life (Ouedraogo et al. 2021). Another reason for this might be the generation gap. Our youngest colleagues currently working as veterinarians are Generation Y (born between 1981 and 2000). Generation Y is more resistant to traditional hierarchy than previous generations and expects higher status, better working conditions and a freer working environment (Kersebohm et al. 2017). Because of these expectations, young veterinarians might have shown more burnout symptoms than their older colleagues (Gen X or Gen Baby Boomer). Here, the concept of healthy‐worker‐survivor bias should also be taken into account. As individuals with high burnout levels leave the profession over time, those who participate in the study are those who are still in the workforce. In other words, a filter naturally forms in favour of employees being healthier, more adaptable and more resilient (Buckley et al. 2015). As the study included employees who have endured burnout, a biased result such as ‘those who are more senior in the profession are less burnt out’ may emerge. It should also be kept in mind that young veterinarians may need experience and time to find more suitable working environments for themselves.

The study found that income level was the strongest predictor of both burnout and job satisfaction. Although it was observed that age, years of professional experience, parenthood, field of study and income level variables had an effect on job satisfaction according to the ANOVA test and independent *t*‐test results, when the effects of the variables were examined separately with regression analysis, it was seen that the income level variable was the only predictor on the level of job satisfaction. It was observed that participants in the higher income group had higher job satisfaction and lower burnout levels, and that as income levels increased, job satisfaction increased and burnout levels decreased. These results are similar to the results of other studies conducted with veterinarians (Kersebohm et al. 2017; Ouedraogo et al. 2021; Jansen et al. 2024). This positive effect of income level on both job satisfaction and burnout may be due to the fact that high‐income individuals feel financially secure. In addition, employees tend to think that the ‘value’ of their work is understood and that they are successful to the extent that they earn a high income (O'Donnell and Mirtcheva‐Broderson 2015). This may have increased job satisfaction and facilitated stress management. Recent studies indicate that higher income levels are often associated with increased job satisfaction, not solely due to financial gain but also because higher‐paying positions frequently offer enhanced job characteristics. These positions often provide greater autonomy, opportunities for skill development and more significant responsibilities, all of which contribute to a more fulfilling work experience. Moreover, increased income can alleviate financial stress, allowing individuals to focus more on their professional growth and job performance (Wu et al. 2015; Katebi et al. 2022; Karasek, 1979). However, it is important to note that although income contributes to job satisfaction, it is not the sole determinant.

The regression analysis and the ANOVA test results indicated that participants who worked fewer hours or participants with more flexible working hours showed lower levels of burnout compared to other participants. The results of this study are parallel to previous studies (Kersebohm et al. 2017; Elkins and Elkins 1987; Shirangi et al. 2013; Frank et al. 1999). Veterinarians are exposed to more problems and more difficult interpersonal interactions as their working hours increase (Wallace 2019). In addition, individuals may feel as if they have lost control of their lives because they cannot spend enough time with their families, friends or non‐work‐related interests (Maslach and Jackson 1981; Wallace 2019). As a result, it is an expected result that long working hours increase burnout levels.

Although the independent *t*‐test results revealed that female participants had significantly higher burnout levels compared to male participants, and that single participants had higher burnout levels compared to married ones, these differences did not remain statistically significant in the multiple regression analysis. This indicates that the associations observed in the bivariate analysis may have been confounded by other factors such as age, parenthood, working hours or income level. When these variables were controlled in the multifactorial model, neither gender nor marital status was found to be an independent predictor of burnout. Thus, it can be suggested that the relationship between these demographic variables (gender, maritual status) and burnout is likely influenced by other demographic and occupational factors such as income level, parenthood and professional experience.

In the present study, it was found that participants who answered ‘yes’ to the question *Did you choose the veterinary profession willingly?* and the question *If you had the chance, would you want to be a veterinarian again?* had higher job satisfaction levels and lower burnout levels compared to participants who answered ‘no’. The study results are consistent with the literature (Özyurt 2004; Babaoğlu et al. 2012). This is important in terms of showing the positive effects of conscious career choice on job satisfaction and burnout. It is reported that students who make conscious career choices have more successful education in terms of enthusiasm for accessing knowledge and skills and graduate more equipped in terms of problem‐solving skills (Akkermans et al. 2018). It is argued that having problem‐solving skills reduces burnout levels and increases job satisfaction (Wallace 2019; Ulum 2019; Morgeson and Humphrey 2006; Derakhshanrad et al. 2019; Özen et al. 2023). When the results of the present study are evaluated with the speculations made in the literature, it can be concluded that those who say they can choose the veterinary profession again have higher problem‐solving skills; therefore, they are more successful in coping with the difficulties and responsibilities they encounter while practicing their profession, and they are positively affected in terms of job satisfaction and burnout.

There are a limited number of studies conducted on the burnout levels of veterinarians in terms of their field of work (Steffey et al. 2023; Gardner and Hini 2006; Rejula et al. 2003). On the basis of the results of these studies, it was reported that veterinarians working in small animal/mixed practices in New Zealand experienced more stress and depression than those working in other fields (Gardner and Hini 2006). In another study that was conducted in Finland (Rejula et al. 2003), the group of participants who showed the highest level of stress was the group consisting of academic veterinarians. Although those who had the highest level of burnout among Finnish veterinarians were those working in the government, veterinarians working in their clinics, that is, veterinarians who owned clinics, and those working in municipalities had lower burnout levels when compared to other fields of work (Rejula et al. 2003). According to the results of the regression analysis and the ANOVA test, municipal veterinarians exhibited the highest levels of burnout. Small animal veterinarians also reported considerably high levels of burnout compared to most other groups. Although the results obtained from small animal clinic operators are parallel to the results presented by Gardner and Hini (2006), the fact that veterinarians working in municipalities showed the highest level of burnout is opposite to the results presented by Rejula et al. (2003). This might have occurred because of the differences in working conditions between Finnish and Turkish veterinarians, or it might be because of the failure of the stray animal rehabilitation project, which was carried out under the responsibility of municipalities in Türkiye for 20 years, making veterinarians the target of criticism. The reason for the high level of burnout among clinician veterinarians might be associated with the effects of high workload, long working hours, urgent and unexpected situations to be dealt with, and the expectations of animal owners, as stated by Hatch et al. (2011). These factors might have created pressure on clinician veterinarians and therefore caused them to show signs of burnout. Another interesting result was that some studies (Hatch et al. 2011; Rejula et al. 2003) reported that academics working in academia and research institutes showed the highest levels of stress and depression compared to veterinarians in other fields of study. In contrast, our study found that both the regression and the ANOVA test results indicated the lowest levels of burnout were observed among academics. The fact that more positive results were obtained from veterinarian academics in Türkiye compared to their Finnish colleagues might be because academics in Türkiye have flexible working hours (Danıştay 2022). It might be argued that the result of this study that flexible working hours positively affected burnout and job satisfaction levels also supports this speculation.

Although the models revealed statistically significant predictors, the *R* ^2^ values remained relatively low (0.19 for both models). This suggests that additional factors beyond the demographic and occupational variables included may significantly influence MBI and MSQ scores. Human outcomes like burnout and job satisfaction are complex and influenced by many unmeasured factors. Possible unmeasured factors may include role conflict, personal characteristics (personality traits, emotional intelligence), coping mechanisms (self‐analytical techniques, counselling or therapy), career advancement opportunities, work‐life balance, job security perceptions and psychological resilience (Maslach and Leiter 2016; Judge et al. 2002; Kagan et al. 2021). Including such variables in future studies could improve the explanatory power of the models.

## 5 Conclusion

As a result of the present study, it was concluded that job satisfaction and burnout levels affect each other in veterinarians. It was also found that veterinarians have a moderate level of job satisfaction in Türkiye Being older, having more years in the profession, being a parent, having a high income and working in academia were associated with high job satisfaction. It was found that those who worked less than 8 h and those who worked with flexible work schedules had lower burnout levels compared to other participants. It was seen that high income strongly increased job satisfaction. It was also found that having chosen the veterinary profession willingly and thinking of wanting to be a veterinarian again if given the chance was positively associated with burnout and job satisfaction.

## 6 Limitations

There might have been an increase in favour of male participants because older veterinarians are generally more male. Second, access bias because of the online nature of the questionnaire (older veterinarians in the country might not use the internet) might have limited the participation. Third, there is no definitive data on the sociodemographic characteristics of veterinarians in Türkiye, such as age, gender or marital status. Therefore, assessments regarding these characteristics are based on estimations (Ankara Chamber of Veterinary Surgeons 2021). Therefore, proportional stratified sampling could not be applied during participant selection, which may have introduced bias towards certain subgroups. For example, although approximately 30%–35% of veterinarians in Türkiye are employed by the Ministry of Agriculture and Forestry (Turkish Veterinary Medical Association 2022), this proportion was 57% among the study participants. This overrepresentation may have caused a sampling bias favouring veterinarians working in the Ministry. Nevertheless, efforts were made to minimize this potential bias by conducting regression analyses in the study. Fourth, the responses of the participants might have been biased because the scales used in the study were self‐reported questionnaires. Finally, as the study was conducted cross‐sectionally, the validity of the findings was limited, and as it was not a longitudinal study, professional burnout and job satisfaction might not have been fully explained. Despite all these limitations, it might be argued that the results of this study reflect the burnout and job satisfaction levels of veterinarians in Türkiye and therefore will be beneficial in creating necessary intervention programs to prevent professional burnout and increase job satisfaction among veterinarians.

## Author Contributions

**Seda Çavuş Alan**: conceptualization (equal), investigation (lead), methodology (equal), formal analysis (lead), writing – review and editing. **Rahşan Özen**: conceptualization (equal), methodology (equal), investigation (support), supervision (equal), writing – review and editing (equal). **Abdullah Özen**: methodology (equal), formal analysis (supporting), validation, supervision (equal), writing – review and editing (equal). **Abdullah Eryol**: conceptualization (equal), investigation (supporting), visualization.

## Ethics Statement

Ethics committee approval was received from Fırat University Social and Human Sciences Research Ethics Committee with the decision number 2023/12 dated 14.06.2023.

## Conflicts of Interest

The authors declare no conflicts of interest.

## Peer Review

The peer review history for this article is available at https://publons.com/publon/10.1002/vms3.70500.

## Supporting information




**Supporting File 1**: vms370500‐sup‐0001‐SuppMat.docx

## Data Availability

The data presented in this study are available on request from the corresponding author.
