# Supplementary material for: A Study on Job Satisfaction and Burnout Levels of Veterinarians in Türkiye
Source: Vet Med Sci. 2025 Jul 18;11(4):e70500. doi: 10.1002/vms3.70500 (PMC12271836; doi:10.1002/vms3.70500)
Supplement: Supplementary file 1 — Supporting File 1: vms370500‐sup‐0001‐SuppMat.docx [file VMS3-11-e70500-s001.docx]

**Investigation of Job Satisfaction and Occupational Burnout Levels of Veterinarians in Türkiye**

Dear colleague,

This research is being conducted by faculty members of the Department of History of Veterinary Medicine and Deontology at Fırat University and Atatürk University Faculty of Veterinary Medicine in order to reveal the relationship between job satisfaction and burnout levels of veterinarians in Türkiye. The ethics committee approval of the study was obtained from the Fırat University Social and Human Sciences Research Ethics Committee with the decision number 22 dated 14.06.2023.

We estimate that the time you will spend for the survey will be approximately 8-10 minutes. Your participation in the research is based on volunteering and you will not encounter any risk in this participation. Similarly, you will not encounter any negativity if you do not participate in the survey or if you give up after agreeing to participate. No information will be requested from you to determine your identity during the research. Your answers will be kept confidential and will only be used by researchers for scientific studies. In order to obtain reliable information from the research results, it is very important to answer the questions sincerely and completely.

IMPORTANT: After filling out the form, please complete the survey by pressing the "send" button at the bottom. Otherwise, your survey answers will not reach us.

If you confirm that you accept this survey form, which is within the scope of an individual research and which you will fill out anonymously, to be used for scientific purposes, to be presented and published in compliance with the rules of confidentiality, without any pressure or coercion, of your own free will, please check the box below.

( )

**Personal dataa**

1. What is your gender?

( ) Female ( ) Male

2. How old are you?

( ) 22-30 ( ) 31-39 ( ) 40-48 ( ) 49-57 ( ) 58 and over age

3. What is your marital status?

( ) Married ( ) Single

4. Do you have children?

( ) Yes ( ) No

5. How many years of professional experience do you have?

( ) 0-5 ( ) 6-10 ( ) 11-15 ( ) 16-20 ( ) 21-25 ( ) 26 and over age

6. What is your field of work?

( ) Pet clinic ( ) Farm animal medicine ( ) Ministry of Agriculture and Forestry ( ) Academic veterinarian ( ) Municipality ( ) Occupational veterinarians ( ) Poultry ( ) Pharmaceutical industry Other- Please specify in writing ( )

7. Did you choose the veterinary profession willingly? ( ) Yes ( ) No

8. If you had a choice, would you still want to be a veterinarian?

( ) Yes ( ) No

9. How would you describe your income level?

( ) Low ( ) Middle ( ) High

10. What are your daily working hours? ( ) less than 8 hours ( ) 8-10 hours ( ) 10-12 hours ( ) Flexible

**Please read each item below and mark the item that is most appropriate for you.**

**Maslach Burnout Inventory**

**(0-Never, 1-very rarely, 2- sometimes, 3-often, 4-always)**

1. I feel emotionally drained from my work
2. I feel used up at the end of the workday
3. I feel fatigued when I get up in the morning and have to face another day on the job
4. Working with people all day is really a strain for me
5. I feel burned out from my work
6. I feel frustrated by my job
7. I feel I’m working too hard on my job
8. Working with people directly puts too much stress on me
9. I feel like I’m at the end of my rope
10. I can easily understand how my recipients feel about things
11. I deal very effectively with the problems of my recipients
12. I feel I’m positively influencing other people’s lives through my work
13. I feel very energetic
14. I can easily create a relaxed atmosphere with my recipients
15. I feel exhilarated after working closely with my recipients
16. I have accomplished many worthwhile things in this job
17. In my work, I deal with emotional problems very calmly
18. I feel I treat some recipients as if they were impersonal ‘objects’
19. I’ve become more callous toward people since I took this job
20. I worry that this job is hardening me emotionally
21. I don’t really care what happens to some recipients
22. I feel recipients blame me for some of their problems

**Minnesota Satisfaction Questionnaire**

**(1-Very dissatisfied, 2- Dissatisfied, 3- Neither, 4- Satisfied, 5- Very satisfied)**

On my present job, this is how I feel about •••

1. Being able to keep busy all the time
2. The chance to work alone on the job
3. The chance to do different things from time to time
4. The chance to be “somebody” in the community
5. The way my boss handles his/her workers
6. The competence of my supervisor in making decisions
7. Being able to do things that don’t go against my conscience
8. The way my job provides for steady employment
9. The chance to do things for other people
10. The chance to tell people what to do
11. The chance to do something that makes use of my abilities
12. The way company policies are put into practice
13. My pay and the amount of work I do
14. The chances for advancement on this job
15. The freedom to use my own judgment
16. The chance to try my own methods of doing the job
17. The working conditions
18. The way my co-workers get along with each other
19. The praise I get for doing a good job
20. The feeling of accomplishment I get from the job
